# Meta-transcriptome Profiling of the Human-*Leishmania braziliensis* Cutaneous Lesion

**DOI:** 10.1371/journal.pntd.0004992

**Published:** 2016-09-15

**Authors:** Stephen M. Christensen, Laura A. L. Dillon, Lucas P. Carvalho, Sara Passos, Fernanda O. Novais, V. Keith Hughitt, Daniel P. Beiting, Edgar M. Carvalho, Phillip Scott, Najib M. El-Sayed, David M. Mosser

**Affiliations:** 1 Department of Cell Biology and Molecular Genetics, University of Maryland, College Park, Maryland, United States of America; 2 Center for Bioinformatics and Computational Biology, University of Maryland, College Park, Maryland, United States of America; 3 Universidade Federal da Bahia Salvador, Bahia, Brazil; 4 Department of Pathobiology, School of Veterinary Medicine, The University of Pennsylvania, Philadelphia, Pennsylvania, United States of America; Ohio State University, UNITED STATES

## Abstract

Host and parasite gene expression in skin biopsies from *Leishmania braziliensis-*infected patients were simultaneously analyzed using high throughput RNA-sequencing. Biopsies were taken from 8 patients with early cutaneous leishmaniasis and 17 patients with late cutaneous leishmaniasis. Although parasite DNA was found in all patient lesions at the time of biopsy, the patients could be stratified into two groups: one lacking detectable parasite transcripts (PT^Neg^) in lesions, and another in which parasite transcripts were readily detected (PT^Pos^). These groups exhibited substantial differences in host responses to infection. PT^Pos^ biopsies contained an unexpected increase in B lymphocyte-specific and immunoglobulin transcripts in the lesions, and an upregulation of immune inhibitory molecules. Biopsies without detectable parasite transcripts showed decreased evidence for B cell activation, but increased expression of antimicrobial genes and genes encoding skin barrier functions. The composition and abundance of *L*. *braziliensis* transcripts in PT^Pos^ lesions were surprisingly conserved among all six patients, with minimal meaningful differences between lesions from patients with early and late cutaneous leishmaniasis. The most abundant parasite transcripts expressed in lesions were distinct from transcripts expressed *in vitro* in human macrophage cultures infected with *L*. *amazonensis* or *L*. *major*. Therefore *in vitro* gene expression in macrophage monolayers may not be a strong predictor of gene expression in lesions. Some of the most highly expressed *in vivo* transcripts encoded amastin-like proteins, hypothetical genes, putative parasite virulence factors, as well as histones and tubulin. In summary, RNA sequencing allowed us to simultaneously analyze human and *L*. *braziliensis* transcriptomes in lesions of infected patients, and identify unexpected differences in host immune responses which correlated with active transcription of parasite genes.

## Author Summary

*Leishmania* spp are intracellular protozoan parasites that replicate primarily within host tissue macrophages. In this paper we simultaneously query host and parasite gene expression in human cutaneous *L*. *braziliensis* lesions. We observe an unexpectedly prominent role for B cells and immunoglobulins in lesions in which actively transcribing parasites reside. We also observe that parasite gene expression is surprisingly conserved among *L*. *braziliensis* lesions, and the genes that are expressed in lesions are not those that have been previously associated with parasite growth *in vitro*. This analysis of parasite and host gene expression in lesions may lead to the identification of new parasite virulence factors and may identify host responses that promote parasite persistence in lesions.

## Introduction

Leishmaniasis is characterized as a spectral disease, with clinical presentations ranging from a self-healing cutaneous form to a visceral form associated with high morbidity and mortality. The immune response to the various species is also spectral in nature, with *L*. *donovani* inducing minimal immune responses or actually inhibiting inflammation and immunity [1], and *L*. *braziliensis* inducing immune activation and immune-mediated pathology [2]. *L*. *braziliensis* is the causative agent of tegumentary leishmaniasis in South America. Approximately 3–5% of patients infected with *L*. *braziliensis* will eventually develop mucocutaneous disease, a disfiguring manifestation involving the nasal and oropharyngeal mucosa [3]. *L*. *braziliensis* infections are typically associated with a strong Th1 response and a positive skin test response to soluble leishmanial antigens characterized by high levels of TNF and IFN-γ [4].

‘Early’ *L*. *braziliensis* lesions frequently present as a small papule that can eventually progress to larger ‘late’ ulcerative lesions, typically containing small numbers of parasites. The more severe mucocutaneous forms of the disease are associated with increased cytokine responses and increased T cell proliferation responses to parasite antigens [4]. The robust immune response to this organism despite low or undetectable numbers of parasites in lesions, and the increased immune responses in mucocutaneous forms of the disease have led to the suggestion that exaggerated Th1 host immune responses contribute to the pathological tissue damage associated with *L*. *braziliensis* infections [5]. Recent studies suggest that a major factor in the development of disease caused by *L*. *braziliensis* is the recruitment of cytolytic CD8+ T cells that promote increased inflammation [6,7].

Immune responses in the skin of infected patients may not be accurately reflected by the type and magnitude of host responses in peripheral blood. Previous studies using immunohistochemistry have described the infiltration of T cells, macrophages, B cells, NK cells, and granulocytes into lesions [8–10]. Some research has implicated CCR2-positive monocytes in parasite killing [11,12], while other studies have described the contribution of the skin micro-environment to the local immune response [13–16]. For these reasons, a meta-transcriptomic profiling of lesions may provide a more complete picture of host and parasite responses during infection.

A microarray-based transcriptomic profiling of skin lesions from patients infected with *L*. *braziliensis* was recently reported [17]. This work uncovered an association between chemokines and cytotoxic T cell responses and disease-associated pathology. We extended this work using RNA-seq to simultaneously analyze host and parasite transcripts in these lesions. This approach allowed the detection of parasite transcripts expressed in the skin of infected patients, and revealed unexpected differences in the host response associated with the detectability of parasite transcripts in lesions. It also revealed that the parasite transcripts most abundantly produced in human lesions could not be predicted by *in vitro* cultivation of parasites in human macrophages.

## Results

### The human host transcriptome in lesions of *L*. *braziliensis* infected patients

**Fig 1:**
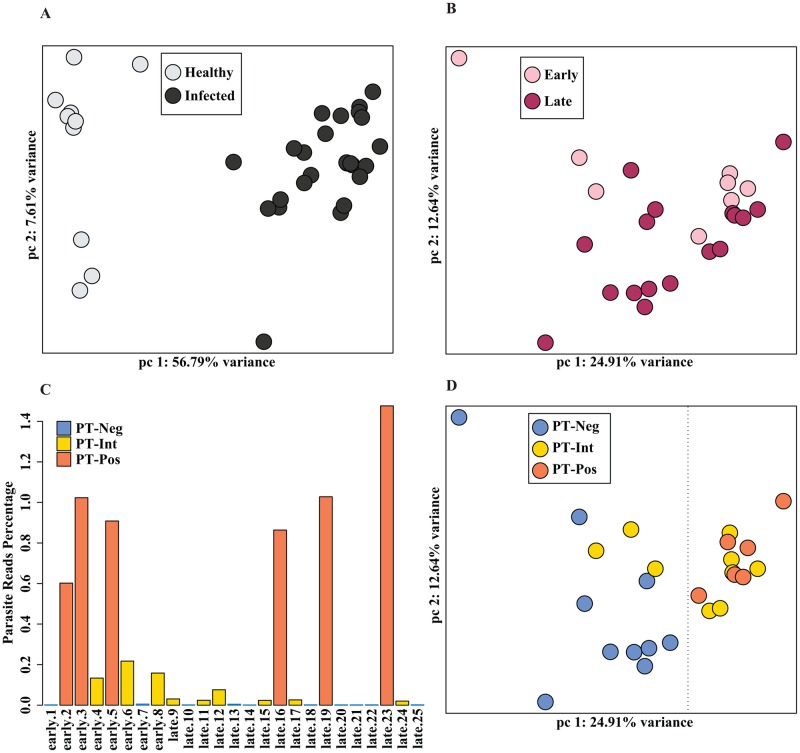
Human and parasite transcriptomes in lesions of *L*. *braziliensis* infected patients. **(1A)** Principal component analysis (PCA) of human transcriptomes from 10 healthy volunteers (light gray) and 25 *L*. *braziliensis*-infected patients (dark gray) is shown. The first two principal components (PC) are displayed on each axis along with the variance (56.79% and 7.61%). **(1B)** Principal component analysis (PCA) plot of human transcriptomes from 8 early-stage (pink), and 17 late-stage (maroon) leishmaniasis patients is shown. The first two principal components (PC) are displayed on each axis along with the variance (24.91% and 12.64%). **(1C)** The percentage of total reads that mapped to the *Leishmania braziliensis* genome from each of the 25 infected patient samples are plotted as bars. The samples containing > 0.5% reads are labeled as parasite transcript positive (PT^Pos^, orange), 0.01–0.5% reads as parasite transcript intermediate (PT^Int^, yellow), and the rest < 0.01% reads as parasite transcript negative (PT^Neg^, blue). **(1D)** A PCA plot of patient samples demonstrates grouping according to parasite transcript levels: PT^Neg^ (blue), PT^Int^ (yellow), and PT^Pos^ (orange). The first two principal components (PC) are displayed on each axis along with the variance (24.91% and 12.64%).

RNA sequencing analysis was performed on biopsies from 10 healthy and 25 *L*. *braziliensis* infected patients. Principal Component Analysis (PCA) (Fig 1A) and hierarchical clustering (S1A Fig) of the human transcriptome data revealed distinct differences between healthy skin and lesions from infected patients, as expected. Lesions are clinically defined as either ‘early’ (small papule, no ulceration, ≤ 30 days illness duration) or ‘late’ (ulcerated lesions, ≥ 30 days illness duration) (S1 Table). Within the group of biopsies from the 25 infected individuals, there were minimal appreciable differences in host responses between patients with early and late cutaneous leishmaniasis (Fig 1B, S2 Table), as previously reported [17]. There was also no separation in host responses as a function of sex (S1B Fig), lesion size (S1C Fig), illness duration (S1D Fig), or age (S1E Fig).

RNA-seq allows the simultaneous analysis of both host and parasite transcriptomes, and we sought to determine whether the presence or absence of detectable parasite transcripts in lesions could impact host responses. Lesions from six of the patients were considered parasite transcript positive (PT^Pos^) when greater than 0.5% of the total reads mapped to the parasite genome (Fig 1C, orange). Ten lesions were considered parasite transcript negative (PT^Neg^) because less than 0.01% of the total reads mapped to the parasite (Fig 1C, blue). This is the same proportion of parasite reads that map non-specifically to highly conserved homologous sequences in the human genome. Nine lesions were considered parasite transcript intermediate (PT^Int^) when percent reads ranged from 0.01–0.2 (Fig 1C, yellow). The number of parasite reads in this group was too low to provide a meaningful analysis of parasite gene expression, and therefore they were excluded from the PT^Pos^ group. By PCA, there was a clear separation in host responses of the PT^Pos^ (Fig 1D, orange) and PT^Neg^ groups (Fig 1D, blue, note dashed line separating blue and orange), with the PT^Int^ group (Fig 1D, yellow) falling in between these two. This separation between groups becomes even more obvious with the addition of the third principal component, accounting for 11.89% of the variance, where there is a clear separation in host responses between PT^Pos^ and PT^Neg^ lesions (S1F Fig). Thus, there is a surprising separation of host responses, depending on the presence or absence of detectable parasite transcripts in lesions, allowing us to correlate host responses with parasite persistence or elimination.

### Host transcriptomic responses in PT^Pos^ and PT^Neg^ samples

**Fig 2:**
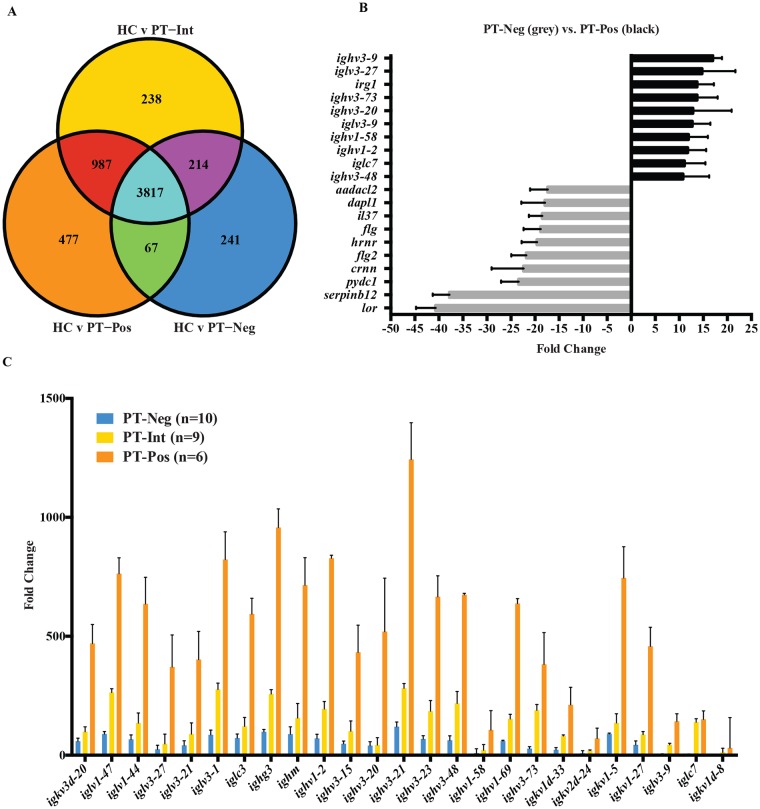
Comparison of human host transcriptomes in lesions with parasite detectable-positive transcripts (PT^Pos^) versus parasite detectable-negative (PT^Neg^) transcripts. **(2A)** A Venn diagram shows differentially expressed genes in three patient populations compared to healthy controls (HC): PT^Pos^ (orange circle), PT^Neg^ (blue circle), PT^Int^ (yellow circle). **(2B).** Shown are the top ten significantly upregulated (black) and downregulated (light gray) host genes when comparing PT^Pos^ to PT^Neg^ (mean plus SEM). **(2C)** The 25 immunoglobulin genes exhibiting the highest differential expression between PT^Neg^ and PT^Pos^ samples are shown. Bars depict the fold change in expression in PT^Neg^ (blue), PT^Int^ (yellow), and PT^Pos^ (orange) samples relative to healthy controls (mean plus SEM).

We found large differences in host transcripts between healthy controls and infected patients, as expected, with 4579 host genes being differentially expressed in lesions relative to normal skin using a cutoff fold change > 2 and *P* value < 0.05 (S3 Table). A total of 3884 differentially expressed genes were common to PT^Neg^ and PT^Pos^, but there was a total of 477 differentially expressed genes unique to PT^Pos^ lesions (Fig 2A, orange circle) and 241 differentially expressed genes unique to PT^Neg^ lesions (Fig 2A, blue circle), relative to healthy controls. The intermediate (PT^Int^) samples shared 3817 differentially expressed genes with PT^Neg^ and PT^Pos^, as well as an additional 987 genes with PT^Pos^ and 214 genes with PT^Neg^. A total of 238 genes showed differential expression unique to the PT^Int^ group (Fig 2A, yellow circle). S4 Table shows direct quantitative comparisons between gene expression in PT^Pos^ and PT^Neg^ lesions, totaling 719 differentially expressed genes. Twenty-five of the top 30 upregulated genes in PT^Pos^ lesions relative to PT^Neg^ lesions encoded immunoglobulin fragments, including 9 of the top 10 genes (Fig 2B, black bars). This group also included CXCL8 (granulocyte migration), IL-21 (B cell proliferation), and granulysin (cellular cytotoxicity). The genes most highly expressed in PT^Neg^ lesions relative to PT^Pos^ lesions included genes involved in skin defenses and epidermal cell development. The top 10 genes in this category included loricrin, filaggrin-1, filaggrin-2, and hornerin, all involved in skin development (Fig 2B, light gray bars).

Although immunoglobulin gene expression in PT^Neg^ lesions increased slightly relative to uninfected healthy skin (Fig 2C, blue), immunoglobulin gene expression in PT^Pos^ lesions was substantially higher (367 fold ± 261 compared to 56 fold ± 32) (Fig 2C, orange). The level of immunoglobulin transcripts in the PT^Int^ group (Fig 2B, yellow) fell between the PT^Pos^ and PT^Neg^ groups (122 fold ± 75). Thus, high immunoglobulin levels in lesions may portend a poor prognosis in this disease, as previously suggested (23).

### Immune response signatures of PT^Pos^ and PT^Neg^ patients

**Fig 3:**
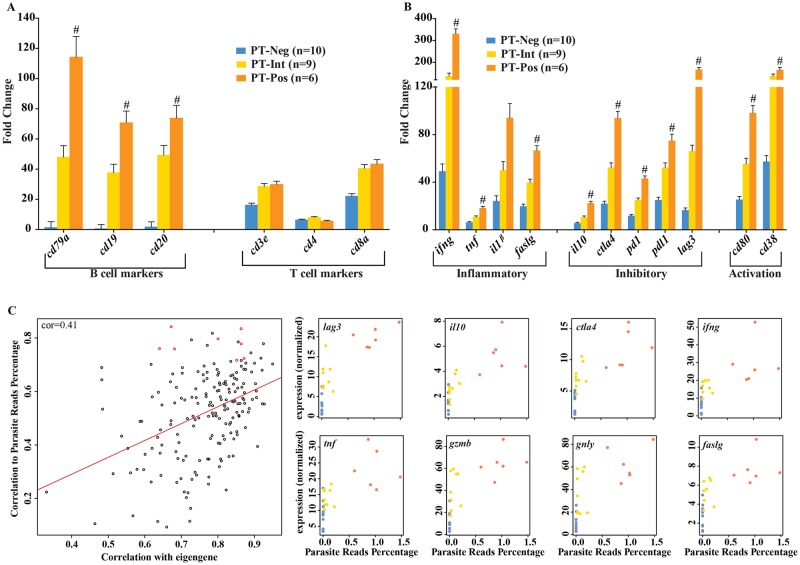
Immune response signatures in parasite transcript positive (PT^Pos^) and negative (PT^Neg^) lesions. **(3A)** Bars represent the fold change of RNA expression of cell-specific markers in PT^Neg^ (blue), PT^Int^ (yellow), and PT^Pos^ (orange) samples, each compared to healthy controls (mean plus SEM). Statistically significant differentially expressed genes, when comparing PT^Pos^ to PT^Neg^ are identified with a # symbol (p<0.05) **(3B)** Bars depict fold changes of transcripts encoding inflammatory cytokines (*ifnγ*, *tnf*, *IL12p40*, *IL-10*, *faslg*), anti-inflammatory and inhibitory signals (*il10*, *ctla4*, *pdcd1*, *cd274*, *lag3*), and activation markers (*cd80*, *cd38*) in lesions of all three classifications compared to healthy controls (mean plus SEM). Statistically significant differentially expressed genes when comparing PT^Pos^ to PT^Neg^ are identified with a # symbol (p<0.05). **(3C)** Shown is the top module (by p-value, see * in S3A Fig) and representative genes from within the module correlated to percent parasite reads using WGCNA analysis. The left plot demonstrates correlation to the module eigengene versus correlation to parasite percent reads for each gene. On the right, plots demonstrate normalized expression (rpkm) versus parasite percent reads for selected genes from within the module (highlighted in red in the left plot).

An assessment of transcripts encoding cell-specific markers pointed to an increase in B cells and their products in the PT^Pos^ lesions. B cell transcripts encoding CD79A, CD19, and CD20, were upregulated in PT^Pos^ lesions, relative to healthy controls (Fig 3A) and relative to PT^Neg^ lesions (indicated by a # symbol). Transcript levels in the PT^Int^ group fell between PT^Pos^ and PT^Neg^ (Fig 3A, yellow). Increases in transcripts encoding B cell markers were not observed when comparing between early and late cutaneous leishmaniasis lesions (S2A Fig). T cell markers (CD3e, CD4, and CD8a) demonstrated no significant differences between PT^Pos^, intermediate and PT^Neg^ lesions (Fig 3A). No significant differences in T cell markers were detected between early and late cutaneous lesions (S2A Fig) as previously reported [17]. Transcripts for inflammatory molecules (IFN-γ, TNF, IL-1β and FASLG), inhibitory molecules (IL-10, CTLA4, PD1, PDL1, and LAG3), and activation markers (CD80 and CD38) were all higher in PT^Pos^ lesions compared to PT^Neg^ lesions (Fig 3B), with PT^Int^ lesions exhibiting intermediate transcript levels (Fig 3B). In contrast to the differences observed between PT^Pos^ and PT^Neg^ lesions, we saw no significant difference in the levels of transcripts for cytokines or signaling molecules in early versus late lesions (S2B Fig).

We used the weighted gene co-expression network analysis (WGCNA) program to cluster human host gene expression through comparison of gene expression profiles using pair-wise correlations. Cluster profiles were then assessed as a function of parasite transcript abundance. This analysis yielded three modules of genes (among a total of 51 modules in the network) whose expression exhibited a significant correlation with parasite transcript abundance (S3A Fig). The most highly correlated module (see * in S3A Fig) contained 100 genes (Fig 3C, left). Eight of the top 14 genes and their normalized expression versus parasite transcript number are shown in Fig 3C, right. These host genes included IL-10, IFN-γ, TNF, granzyme B and fas ligand. All of the genes in this module exhibited a higher expression in PT^pos^ lesions (Fig 3C, orange dots). The two additional modules that also showed a significant correlation between host gene expression and parasite transcript numbers contained immunoglobulin transcripts, chemokine genes, cytokines and growth factors (S3 Fig).

### Functional interactions and distinct clustering among differentially expressed genes

**Fig 4:**
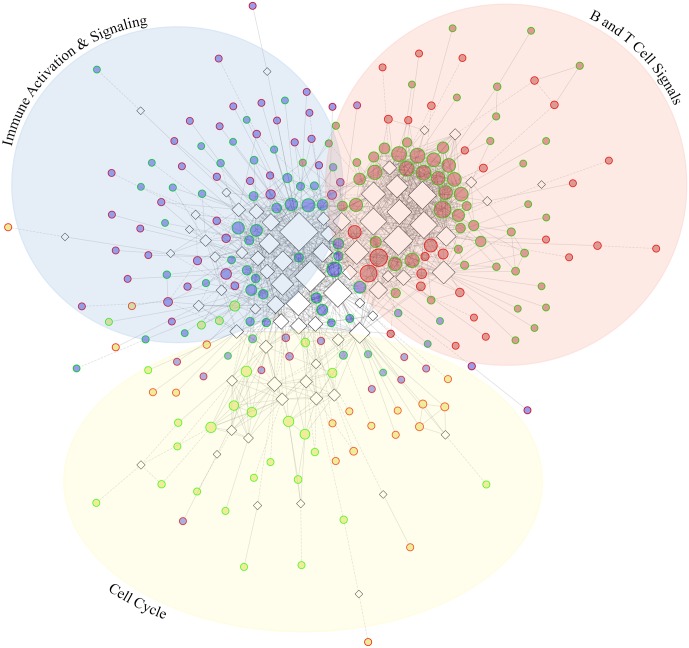
Functional interactions among differentially expressed genes. **(4A)** A group of 237 of the 719 differentially expressed genes between PT^Neg^ and PT^Pos^ (circular nodes) along with 75 associated linker genes (diamond nodes, black border) show numerous functional interactions (edges). Node size is relative to the number of interactions. Gene clusters are depicted by color (blue, red, and yellow) and direction of differential expression of genes within the clusters is depicted by node border, with green borders designating upregulated and red borders designating downregulated genes. Edges represent numerous known (solid line) and predicted (dashed line) functional interactions.

The Reactome FI plugin for Cytoscape was used to observe known interactions between the 719 genes that were differentially expressed between PT^Neg^ and PT^Pos^ (S4 Table). An additional 149 linker genes, known to influence or connect multiple genes in the gene set, were included in this analysis. A network of 558 genes generated numerous clusters showing a dense interaction of genes differentially expressed between PT^Neg^ and PT^Pos^. When clustering genes by functional interactions from the Reactome database, the top three clusters highlight specific immune and cellular pathways (Fig 4). The largest cluster (Fig 4, blue nodes) consists of 137 genes associated with immune cell activation, costimulation, and cytokine and chemokine signaling, including TNF, IL-10, and multiple C-C motif chemokines (S4A Fig). Gene regulation in this category is associated with NF-κβ, CREB/STAT3, JUN, and SP1 signaling. The second largest cluster (Fig 4, red nodes) includes 103 genes involved in B and T cell activation and inhibition, regulated by SYK, SRC, FYN, PLCG1 and 2, and PTPN11 (S4B Fig). Cluster 3 (Fig 4, yellow nodes) includes 72 genes associated with cell growth and proliferation signals (S4C Fig). The transcriptomic differences between PT^Pos^ and PT^Neg^ patients demonstrate the potential of these clusters to lead to the identification of new drug targets for treating cutaneous leishmaniasis.

### Identification of *L*. *braziliensis* transcripts in lesions

**Fig 5:**
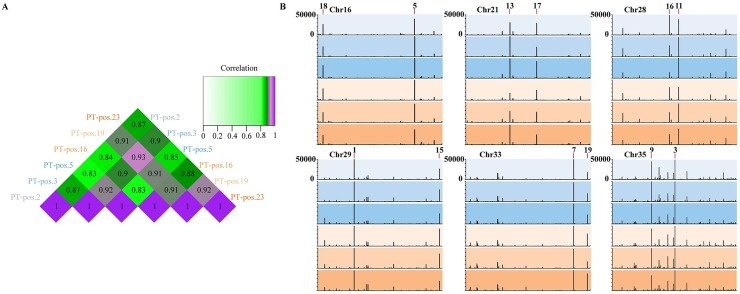
*L*. *braziliensis* gene expression in human lesions. RPKM values of all samples were scaled to the sample with the highest total normalized reads. **(5A)** A heatmap demonstrates Pearson correlation of parasite gene expression of the top 50 parasite genes in the six PT^Pos^ samples. **(5B)** A representation of six parasite chromosomes (16, 21, 28, 29, 33, 35) shows the RPKM levels for each PT^Pos^ patient, early patients depicted in blue hues, late patients in brown hues. RPKM is depicted on the y axis (0 to 50,000), and genes that appear in the 20 most-highly expressed parasite genes are labeled.

We analyzed parasite gene expression in the six PT^Pos^ patient samples and despite substantial differences in lesion size and duration (three early and three late cutaneous PT^Pos^ lesions), there was a high degree of uniformity in *L*. *braziliensis* transcript expression in all six patient lesions. Pearson correlation analysis of the top 50 parasite genes in each sample quantitatively demonstrated the similarity between samples, and ranged from 0.83 to 0.92 (Fig 5A). The 20 most highly-expressed parasite transcripts by average RPKM, listed in Table 1, were fairly randomly dispersed across the parasite genome (S5 Fig). Six of the parasite chromosomes are illustrated in Fig 5B and the parasite gene expression (in RPKM) is shown by vertical lines in the 6 patients (each patient designated by a different color intensity). Again, there is remarkable uniformity in parasite gene expression from patient to patient. Within these six chromosomes, 12 of the top 20 parasite genes (by RPKM) are noted, including cysteine peptidases, cysteine synthase, a proteasome subunit, and various hydrolase-like and hypothetical proteins.

**Table 1:** Top 20 *L*. *braziliensis* genes expressed in detectable-positive lesions as ranked by RPKM.

	ID	Probable function	Average expression(RPKM)
1	LbrM.29.0850	CPC cysteine peptidase, Clan CA, family C1, Cathepsin B-like	107886
2	LbrM.24.2240	hypothetical protein: Glycerophosphoryl phosphodiesterase family	86675
3	LbrM.35.3820	cysteine synthase	85691
4	LbrM.32.0100	protein transport protein SEC13, putative	66806
5	LbrM.06.0120	proteasome beta 6 subunit, putative,20S proteasome beta 6 subunit, putative	58512
6	LbrM.16.1390	hypothetical protein, conserved	55259
7	LbrM.33.3110	D-alanyl-glycyl endopeptidase-like protein,cysteine peptidase, Clan CA,family C51, putative	54840
8	LbrM.26.0610	hypothetical protein, conserved	53296
9	LbrM.18.1250	hypothetical protein:Zinc finger, C3HC4 type (RING finger) containing protein	51205
10	LbrM.35.2380	hypothetical protein, HAD family hydrolase	50482
11	LbrM.28.1740	hydrolase, alpha/beta fold family, putative	43445
12	LbrM.19.1410	hypothetical protein, conserved	41752
13	LbrM.21.0730	metallo-beta-lactamase family-like protein	37552
14	LbrM.34.0990	casein kinase I, putative	35123
15	LbrM.29.2850	inosine-adenosine-guanosine-nucleoside hydrolase, putative	30813
16	LbrM.28.1490	haloacid dehalogenase-like hydrolase-like protein	28703
17	LbrM.21.1170	hypothetical protein, conserved	28426
18	LbrM.16.0080	Hypothetical protein, p46 protein	26611
19	LbrM.21.2150	beta tubulin	25250
20	LbrM.33.3470	cation transporter, putative	24930

All 20 of the most highly expressed parasite genes were expressed in all six patients, regardless of whether the lesions were early or late (S5 Fig). The top parasite transcripts (Table 1) consisted of cysteine peptidases, a phosphodiesterase, as well as kinases and transport proteins. The presence of hypothetical and putative proteins in this list illustrates our lack of understanding of parasite gene expression during natural infections. Several amastin family genes and known virulence factors including GP63, heat-shock proteins 70 and 83, and cysteine peptidases appear in the top 100 observed transcripts (S5 Table).

Our recent work demonstrated the benefits of dual-transcriptomic analyses, using *in vitro* infection of macrophages with *L*. *major* and *L*. *amazonensis* to observe expression pattern changes in the host cell and parasite gene expression, ranging from 4 to 72 hours [18]. To determine whether the *L*. *braziliensis* transcripts identified in lesions were the most highly expressed parasite genes expressed in vitro, we compared the top 40 *L*. *braziliensis* transcripts identified in lesions (ranked by RPKM), to the homologous genes expressed by *L*. *amazonensis* or *L*. *major* 72 hours after an *in vitro* infection of human macrophages, as previously described by us [18] (S6A Fig). A comparison of homologous genes by rank showed that more than 60% of the most highly expressed *L*. *braziliensis* genes *in vivo* (S6A Fig, red) did not match the most highly expressed parasite genes in cultivated macrophages following *in vitro* infection with *L*. *amazonensis* (S6A Fig, green) or *L*. *major* (S6A Fig, blue). Only 15 of the top 40 genes expressed by *L*. *braziliensis* during *in vivo* infections appeared in the top 40 *L*. *major* genes expressed *in vitro*, and only 5 of the top 40 genes appeared in the top 500 *L*. *amazonensis* genes expressed *in vitro*. The converse of matching the most highly expressed genes was also true. The most highly expressed *L*. *amazonensis* genes expressed *in vitro* (S6B Fig, green) were not highly expressed by *L*. *braziliensis in vivo* or by *L*. *major in vitro* (S6B Fig, red and blue). A direct comparison to *L*. *braziliensis* genes expressed *in vitro* was not possible because RNA-seq analysis of *L*. *braziliensis* transcripts following *in vitro* infection of human macrophages has not been performed. This work is underway. Therefore, definitive conclusions regarding differences in gene expression between in vitro and in vivo infections cannot be made. However the results from this inter-species comparison suggests that the transcripts identified in *L*. *braziliensis* lesions were not simply the most highly expressed leishmanial genes, but rather might be transcripts involved in promoting parasite persistence in the lesion microenvironment.

## Discussion

By applying RNA sequencing technology to skin biopsy material, we were able to capture in detail the transcriptomes of both the parasite and the human host during *L*. *braziliensis* localized cutaneous infection. This analysis allowed us to examine host responses as a function of parasite persistence. *L*. *braziliensis* infections have often been described as having low parasite numbers in lesions [19]. This is consistent with our observation that lesions from 10 of the 25 patients had no detectable parasite transcripts in them at the time of biopsy. This lack of detectable transcripts is consistent with an active elimination of parasites by the host. In contrast, six of the 25 patients had ample evidence of high confidence parasite transcripts in lesions (averaging nearly 1M parasite reads/sample; see S1 Table). The presence of these transcripts indicates that viable parasites were persisting in lesions and continuing to produce transcripts that could contribute to their survival. Therefore, we compared host immune responses in lesions where parasites were producing detectable transcripts (PT^Pos^) to those where parasite transcripts were undetectable (PT^Neg^).

The progression of *L*. *braziliensis* disease has been well-studied, and the small nodules of early tegumentary leishmaniasis typically progress to the larger sometimes necrotic lesions associated with late cutaneous leishmaniasis [20]. Studies have been done to examine differences in immune responses between these two groups [21], and comparisons with regard to treatment efficacy between early and late disease have also been made [19,22]. A surprising result from the previous transcriptomic comparison between early and late *L*. *braziliensis* lesions was that the host immune response was initiated early during infection and persisted throughout the course of the disease [17]. Our RNA-seq confirms this previous observation. We could not separate the host response during early and late tegumentary leishmaniasis by Principal Component Analysis (Fig 1). However, when these responses were stratified by the presence or absence of parasite transcripts, a clear separation could be achieved between the host responses in lesions where parasite transcripts were detectable (PT^pos^) versus those where they were not detectable (PT^neg^). We chose to contrast those two groups with an eye to understanding host responses that may be associated with parasite persistence or parasite elimination.

One of the surprising observations from this work was the degree to which B cell transcripts were associated with lesions containing detectable levels of parasite transcripts. Of the top 100 genes that were differentially upregulated in PT^Pos^ lesions relative to healthy controls, 80 encoded immunoglobulin-related transcripts. Lesions in which parasite transcripts were not detectable (PT^Neg^) also showed some evidence of B cell activation, but not to the same extent. The quantity of immunoglobulin transcripts in PT^Pos^ lesions was higher than PT^Neg^ lesions (Fig 2C), as were the number of B cells as judged by transcripts encoding CD79A, CD19, and CD20 (Fig 3A). One interpretation of these results is that the persistence of actively transcribing parasites in the lesion prolongs the host immune response. However, we previously reported that in human visceral leishmaniasis, high levels of IgG were associated with on-going disease, and that IgG levels decreased as cell mediated immunity developed following treatment [23]. Furthermore, addition of parasite-specific IgG to *L*. *major*-infected J_H_ mice exacerbated disease, increasing lesion size and inducing IL-10 production [23]. These previously published observations, along with the present association of high immunoglobulin transcripts in PT^Pos^ lesions, suggest that B cells and host IgG may be strong contributors to parasite persistence.

We and others previously demonstrated that IL-10 could contribute to parasite survival, and a correlation between IgG levels and IL-10 production was identified in visceral leishmaniasis [23,24]. The present studies extend this correlation to American tegumentary leishmaniasis, and demonstrate that PT^pos^ lesions had higher levels of IL-10 than PT^Neg^ lesions (Fig 3B). The association between parasite survival and cytokine production may be more complex than originally perceived, however, since PT^Pos^ lesions also expressed higher levels of IFN-γ and TNF, two cytokines that have well-established roles in classical macrophage activation and parasite elimination.

In addition to an in depth look at the host response, RNA-seq also provides a picture of the parasite transcriptome during infection. Viewing parasite gene expression within the lesion provided some interesting surprises. First and foremost, the uniformity of parasite gene expression across all six patients, given the differences in lesion size, duration, and degree of necrosis among them was unexpected. It is possible that the most highly expressed parasite genes in lesions have the potential to contribute to parasite persistence. The identification of these gene products may lead to new candidates for vaccine development or new targets for diagnosis. In fact, recently published work from Khare et al. [25] targets the conserved kinetoplastid parasite proteasome, of which we observed a subunit highly expressed by *L*. *braziliensis* in patient lesions (Table 1). Also of note is that 8 of the 20 most highly expressed parasite genes encode “hypothetical proteins” of unknown function. The identification and characterization of these proteins may shed new light on how this parasite establishes infection, persists within mammalian cells, or escapes these cells to spread disease. We tested whether the parasite transcripts detected *in vivo* were a reflection of the most abundant transcripts expressed by amastigotes growing in human macrophages *in vitro*. The top 40 transcripts detected in lesions were different from the transcripts most highly expressed *in vitro*, and there was less than 40% (*L*. *major*) or essentially no (*L*. *amazonensis*) overlap between parasite gene expression in cultivated cells and in lesions. Although the parasite species and time post-infection may confound these findings, we believe that a contrast of the *Leishmania* genes expressed *in vivo* versus *in vitro* provides a useful baseline for future comparisons.

We also observed a correlation between the prevalence of parasite transcripts in the lesion and the expression of a subset of host immune response genes. Using the weighted gene co-expression network analysis program (WGCNA), we identified several key host response genes whose expression increased in lesions containing high levels of parasite transcripts (Fig 3C). IL-10 has been previously associated with disease progression, so perhaps its inclusion in this category may not have been unexpected. However the association of TNF, granzyme B, and IFN-γ with parasite persistence was not expected. These observations may indicate that immunopathology is a driving factor in *L*. *braziliensis* infections. The second and third most significant modules (S3 Fig) correlate with several genes involved in B cell responses, immunoglobulin production and T cell interactions with parasite transcriptional activity. These observations are consistent with the hypothesis that B cells and IgG production may contribute to parasite survival.

## Methods

### Ethics statement

This study was conducted according to the principles specified in the Declaration of Helsinki and under local ethical guidelines and this study was approved by the Ethics Committees of the Federal University of Bahia (Salvador,Bahia, Brazil)(010/10), University of Maryland (College Park)(395840–3) and the University of Pennsylvania IRB (Philadelphia, Pa)(813390). All patients provided written informed consent for the collection of samples and subsequent analysis.

### Patients and procedures

All cutaneous leishmaniasis (CL) patients were seen at the health post in Corte de Pedra, Bahia, Brazil, an area endemic to *L*. *braziliensis*. Diagnosis consisted of visual confirmation of a lesion characteristic of CL and parasite DNA detection and/or a positive delayed-type hypersensitivity response to *Leishmania* antigen. Biopsies were collected at the border of the lesions using a 4 mm punch before therapy. Patients consisted of 15 males and 10 females with illness duration ranging from 15 to 90 days and lesion sizes ranging from 4–960 mm^2^ (S1 Table). Healthy (uninfected) skin samples were taken from volunteers living in a non-endemic area without a history of leishmaniasis, as described [17].

### RNA isolation and cDNA library preparation

Samples were placed in RNA later and homogenized using a rotor-stator. Total RNA was isolated using the RNeasy Plus Kit from Qiagen. RNA integrity was assessed using an Agilent 2100 bioanalyzer. Poly(A)^+^-enriched cDNA libraries were generated using the Illumina TruSeq Sample Preparation kit (San Diego, CA) and checked for quality and quantity using bioanalyzer and qPCR (KAPA Biosystems).

### RNA-seq data generation, pre-processing, and quality trimming

Paired end reads (100 bp) were obtained using the Illumina HiSeq 1500 platform. Trimmomatic [26] was used to remove any remaining Illumina adapter sequences from reads and to trim bases off the start or the end of a read when the quality score fell below a threshold of 20. Sequence quality metrics were assessed using FastQC [http://www.bioinformatics.babraham.ac.uk/projects/fastqc/].

### Mapping cDNA fragments to the reference genome, abundance estimation, and data normalization

TopHat (v 2.0.13) [27] was used to align reads to the applicable genome(s) with each genome alignment performed independently. Reads from healthy, early infection, and late infection skin samples were aligned to the human genome (v. hg19/GRCh37) obtained from the UCSC genome browser (http://genome.ucsc.edu). Reads from early infection and late infection samples were additionally aligned to the *L*. *braziliensis* (v. MHOM/BR/75M2904, Sanger Institute) genome obtained from the TriTrypDB database (www.tritrypdb.org). Two mismatches per read were permitted (default TopHat parameter) and reads were allowed to map only to a single locus (TopHat option –g 1). Additionally, gene model annotations were provided for the mapping (TopHat option –G) with limitations on the identification of novel splice junctions (TopHat option –no-novel-juncs). The abundance of reads mapping to each gene feature in the aligned genome was determined using HTSeq [28]. Each resulting count table was restricted to protein-coding genes (20,956 genes for human and 8,556 genes for *L*. *braziliensis*). Non-expressed and weakly expressed genes, defined as having less than 1 read per million in n of the samples, where n is the size of the smallest group of replicates [29] (here n = 8 for both human and parasite), were removed prior to subsequent analyses, resulting in count tables of 15,256 and 8,556 genes for human and *L*. *braziliensis*, respectively.

### Global data assessment, visualization and differential expression analysis

Samples were classified as PT^Pos^ and PT^Neg^ based on the percentage of reads mapping to the *L*. *braziliensis* genome. Parasite detectable-positive samples were defined as those with more than 0.5% of reads mapping to the parasite genome (see Fig 1C). In the six PT^pos^ samples, the average proportion of reads that mapped to the parasite genome was 0.98% (see Fig 1C), for an average of 867,489 parasite reads per tissue sample sequenced to an average depth of 88 million reads (S1 Table). The 10 PT^Neg^ samples were defined as those with fewer than 0.01% reads mapping to the parasite; the proportion of reads in the PT^Neg^ samples that mapped to the parasite genome was not different than the 10 healthy controls. We designated nine additional samples as PT^Int^ because they expressed low levels of transcripts (between 0.01–0.2% of total reads) that could only tentatively be assigned to the parasite. The number of parasite reads in this group was too low to provide a meaningful analysis of parasite gene expression and therefore they were excluded from the PT^Pos^ group. Quantile normalization was applied to all samples [30] and data were log2-transformed. Multiple approaches were used to evaluate replicates and to visualize the relationships between samples, including Pearson correlation and Principal Component Analysis (PCA). Limma (a Bioconductor package) was used to conduct differential expression analyses [31]. The voom module was used to transform the data based on observational level weights derived from the mean-variance relationship prior to statistical modeling [32]. Pairwise contrasts were done within limma to identify differentially expressed (DE) genes between conditions. Genes with a Benjamini-Hochberg (BH) multiple-testing adjusted *P* value of < 0.05 were defined as differentially expressed. Components of our statistical pipeline, named cbcbSEQ, can be accessed on GitHub (https://github.com/kokrah/cbcbSEQ/).

## Supporting Information

S1 Fig **(S1A)** Panel A shows a heatmap enriched pathways (using GSEA) comparing healthy controls (gray line, top) and leishmaniasis patients (orange line). 680 pathways showed ≥2 fold differences that were clustered hierarchically. **(S1B)** Principal component analysis (PCA) plot of human transcriptomes from 15 female (light gray), and 10 male (dark gray) leishmaniasis patients. The first two principal components (PC) are displayed on each axis along with the variance (24.91% and 12.64%). **(S1C)** Principal component analysis (PCA) plot of human transcriptomes from 25 leishmaniasis patients, colored by lesion size. The first two principal components (PC) are displayed on each axis along with the variance (24.91% and 12.64%). **(S1D)** Principal component analysis (PCA) plot of human transcriptomes from 25 leishmaniasis patients, colored by illness duration. The first two principal components (PC) are displayed on each axis along with the variance (24.91% and 12.64%). **(S1E)** Principal component analysis (PCA) plot of human transcriptomes from 25 leishmaniasis patients, colored by patient age. The first two principal components (PC) are displayed on each axis along with the variance (24.91% and 12.64%). **(S1F)** 3-D Principal component analysis (PCA) plot of human transcriptomes from 25 leishmaniasis patients, colored by parasite transcript status. Blue = PT^Neg^, Yellow = PT^Int^, Orange = PT^Pos^. The first three principal components (PC) are displayed on each axis (PC1: 24.91%, PC2: 12.64%, PC3: 11.89%).
(TIF)

S2 Fig RNA expression signatures of immune responses comparing early and late-stage cutaneous lesions. Bars represent the fold change (mean plus SEM) of RNA expression of cell-specific markers in early (light grey) and late (dark grey) cutaneous samples, each compared to healthy patients, showing **(A)** infiltration of B cell (*cd79a*, *cd19*, *cd20*) and T cell (*cd3e*, *cd4*, *cd8a*) biomarkers, and **(B)** increased inflammatory (*ifnγ*, *tnf*, *IL12p40*, *IL-10*, *faslg*), anti-inflammatory and inhibitory signals (*il10*, *ctla4*, *pdcd1*, *cd274*, *lag3*), and activation markers (*cd80*, *cd38*) in lesions with no difference observed between early and late. NS = not significant.
(TIF)

S3 Fig Module correlation to parasite percent reads using WGCNA analysis. **(A)** Heatmap comparing the module eigengene relationship to the trait of percent parasite reads. Heatmap color indicates correlations of -1 (blue) to +1 (red) and p-values are shown in parentheses. Modules are sorted top to bottom by significance and each module is represented by a different color block. The three most significant modules are indicated in red type with the most significant module, indicated by a *, which is explored further in Fig 3C. **(B)** The second (light yellow, top) and third (cyan, bottom) module (by p-value) and representative genes from within the module correlated to percent parasite reads using WGCNA analysis. The left plots demonstrate module membership versus correlation to parasite percent reads for each gene. On the right, plots demonstrate normalized expression (rpkm) versus parasite percent reads for selected genes from within the modules (highlighted in red in the left plots).
(TIF)

S4 Fig Functional interactions among differentially expressed genes. The largest three clusters consisting of 137 (A), 103 (B), and 72 (C) genes show numerous functional interactions (edges). Gene clusters are depicted by color and direction of differential expression is depicted by node border, with green borders designating upregulated and red borders designating downregulated genes. Node size indicates the number of interactions. (A) Network of genes associated with immune cell activation, costimulation, and cytokine and chemokine signaling, including TNF, IL-10, and multiple C-C motif chemokines regulated by NFκβ, CREB/STAT3, JUN, and SP1 signaling. (B) Network of 103 genes involved in B and T cell activation and inhibition, regulated by SYK, SRC, FYN, PLCG1 and 2, and PTPN11. (C) Interaction of 72 genes associated with cell growth and proliferation signals.
(TIF)

S5 Fig Parasite gene expression. Representation of the *L*. *braziliensis* genome with the outermost ring (labeled 1–35) showing each individual chromosome. The subsequent concentric circles represent each of the six PT^Pos^ patient samples (3 early cutaneous shown in blue, 3 late cutaneous in orange). Vertical lines designate the relative expression levels of *L*. *braziliensis* gene expression in RPKM with the top 20 most highly-expressed genes marked by a red line, and numbered from 1–20. Chromosomes labeled in bold are viewed closer in Fig 5.
(TIF)

S6 Fig *L*. *braziliensis* gene expression in lesions and homologous gene expression by *L*. *amazonensis* and *L*. *major* following *in vitro* infection of human macrophages. RPKM values of all samples were scaled to the sample with the highest total normalized reads. **(A)** The top 40 *L*. *braziliensis* genes (red) expressed *in vivo* as ranked by average RPKM (left axis) matched to the ranking of their homologous genes in *L*. *major* (blue, left axis, 1–50+) and *L*. *amazonensis* (green, right axis, 1–500+) expressed during *in vitro* infection of human macrophages at 72 hours post-infection. **(B)** The top 40 *L*. *amazonensis* genes (green) expressed during *in vitro* infection of human macrophages at 72 hours post-infection as ranked by average RPKM (left axis) matched to the ranking (1–500+) of their homologous genes in *L*. *braziliensis* (red, right axis) expressed during *in vivo* and *L*. *major* (blue, right axis) expressed during *in vitro* infection of human macrophages at 72 hours post-infection.
(TIF)

S1 Table Experimental design, phenotypic data, and accession numbers. (XLSX)

S2 Table Differentially expressed genes comparing early and late cutaneous lesions (p<0.05, FC>2). (XLS)

S3 Table Differentially expressed genes comparing healthy controls and infected patients (p<0.05, FC>2). (XLS)

S4 Table Differentially expressed genes comparing PT^Pos^ and PT^Neg^ patients (p<0.05, FC>2). (XLSX)

S5 Table Top 100 *L*. *braziliensis* genes by expression level (RPKM). (XLSX)
