# Correction: Meta-transcriptome Profiling of the Human-*Leishmania braziliensis* Cutaneous Lesion

**DOI:** 10.1371/journal.pntd.0005588

**Published:** 2017-05-03

**Authors:** Stephen M. Christensen, Laura A. L. Dillon, Lucas P. Carvalho, Sara Passos, Fernanda O. Novais, V. Keith Hughitt, Daniel P. Beiting, Edgar M. Carvalho, Phillip Scott, Najib M. El-Sayed, David M. Mosser

There are errors in Table 1. A computational error caused incorrect gene length values to be applied to the parasite RPKM calculations, resulting in changes in the ranking of which parasite genes were most highly expressed by RNA sequencing. A corrected Table 1 is provided here.

**Table 1:** Top 20 *L*. *braziliensis* genes expressed in detectable-positive lesions as ranked by RPKM.

Gene Description (IDs)	*L*. *braziliensis* RPKM
amastin-like surface protein, putative (LbrM.08.1060/08.300/08.1130)	38295
L-ribulokinase, putative (LbrM.35.0100)	11184
amastin-like surface protein, putative (LbrM.18.0460/08.0310/08.290)	7270
histone H2B (LbrM.09.1400)	6390
amastin-like surface protein, putative (LbrM.08.1100/20.4300)	6164
kinesin, putative (pseudogene) (LbrM.20.1090)	5013
unspecified product (LbrM.24.1400)	4973
tagatose-6-phosphate kinase-like protein (LbrM.02.0030)	4232
amastin-like protein (LbrM.08.1030)	3867
histone H1 (LbrM.18.1510)	3827
amastin-like surface protein, putative (LbrM.20.0790)	3778
hypothetical protein, conserved (LbrM.20.3230)	3753
amastin-like protein (LbrM.08.0670)	3727
heat-shock protein hsp70, putative (LbrM.28.2990)	3676
beta tubulin (LbrM.21.2150)	3620
amastin-like surface protein (LbrM.18.0470)	3567
histone H2A (LbrM.21.1140)	3342
amastin-like surface protein, putative (LbrM.20.1080)	3321
histone H2A, putative (LbrM.21.1160)	3192
histone H1, putative (LbrM.27.1290)	3145
