# Supplementary figures and images for: Meta-transcriptome Profiling of the Human-Leishmania braziliensis Cutaneous Lesion
Source: PLoS Negl Trop Dis. 2016 Sep 15;10(9):e0004992. doi: 10.1371/journal.pntd.0004992 (PMC5025153; doi:10.1371/journal.pntd.0004992)

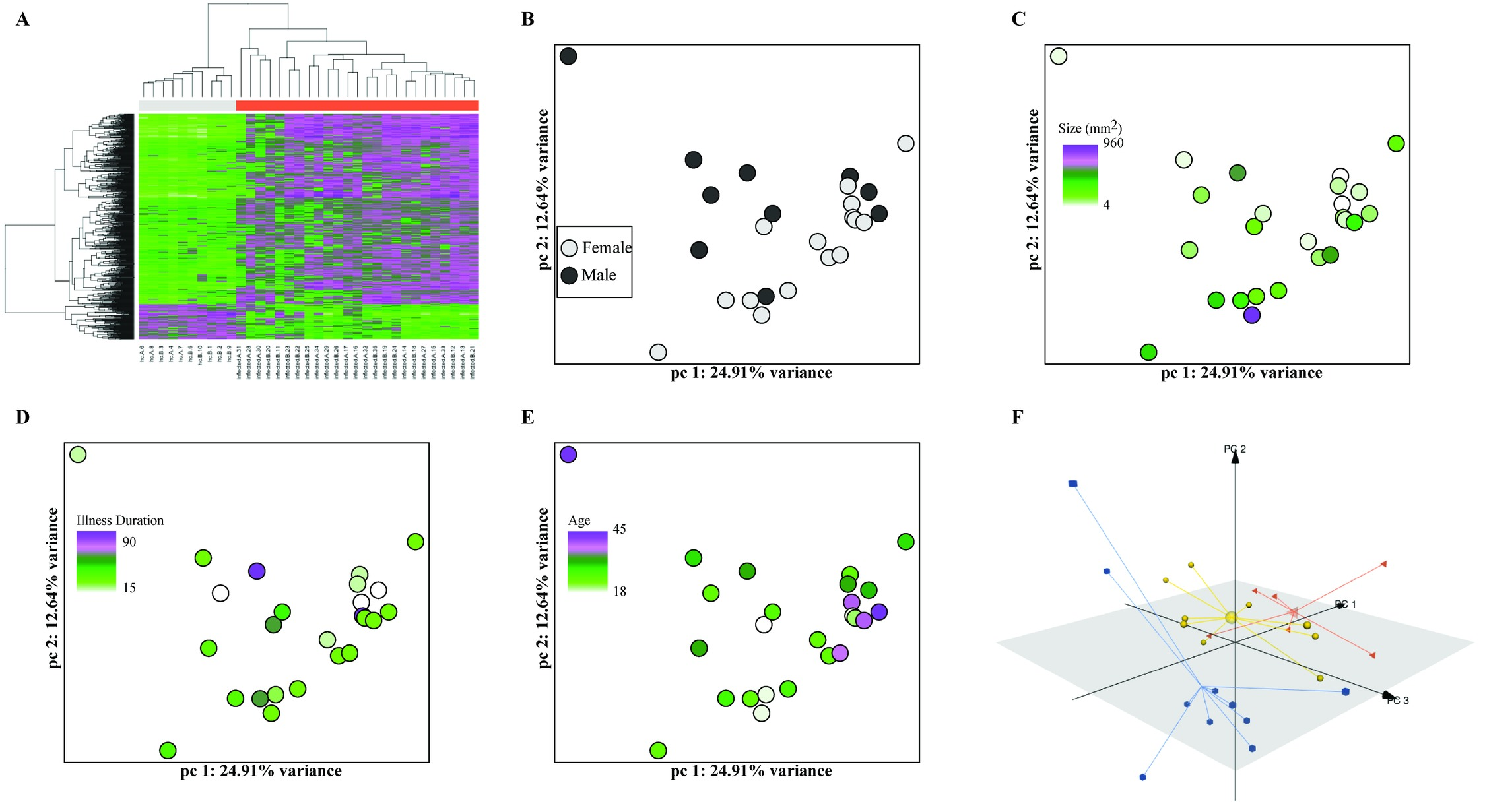

Supplement: S1 Fig — (S1A) Panel A shows a heatmap enriched pathways (using GSEA) comparing healthy controls (gray line, top) and leishmaniasis patients (orange line). 680 pathways showed ≥2 fold differences that were clustered hierarchically. (S1B) Principal component analysis (PCA) plot of human transcriptomes from 15 female (light gray), and 10 male (dark gray) leishmaniasis patients. The first two principal components (PC) are displayed on each axis along with the variance (24.91% and 12.64%). (S1C) Principal component analysis (PCA) plot of human transcriptomes from 25 leishmaniasis patients, colored by lesion size. The first two principal components (PC) are displayed on each axis along with the variance (24.91% and 12.64%). (S1D) Principal component analysis (PCA) plot of human transcriptomes from 25 leishmaniasis patients, colored by illness duration. The first two principal components (PC) are displayed on each axis along with the variance (24.91% and 12.64%). (S1E) Principal component analysis (PCA) plot of human transcriptomes from 25 leishmaniasis patients, colored by patient age. The first two principal components (PC) are displayed on each axis along with the variance (24.91% and 12.64%). (S1F) 3-D Principal component analysis (PCA) plot of human transcriptomes from 25 leishmaniasis patients, colored by parasite transcript status. Blue = PTNeg, Yellow = PTInt, Orange = PTPos. The first three principal components (PC) are displayed on each axis (PC1: 24.91%, PC2: 12.64%, PC3: 11.89%). (TIF) [file pntd.0004992.s001.tif]

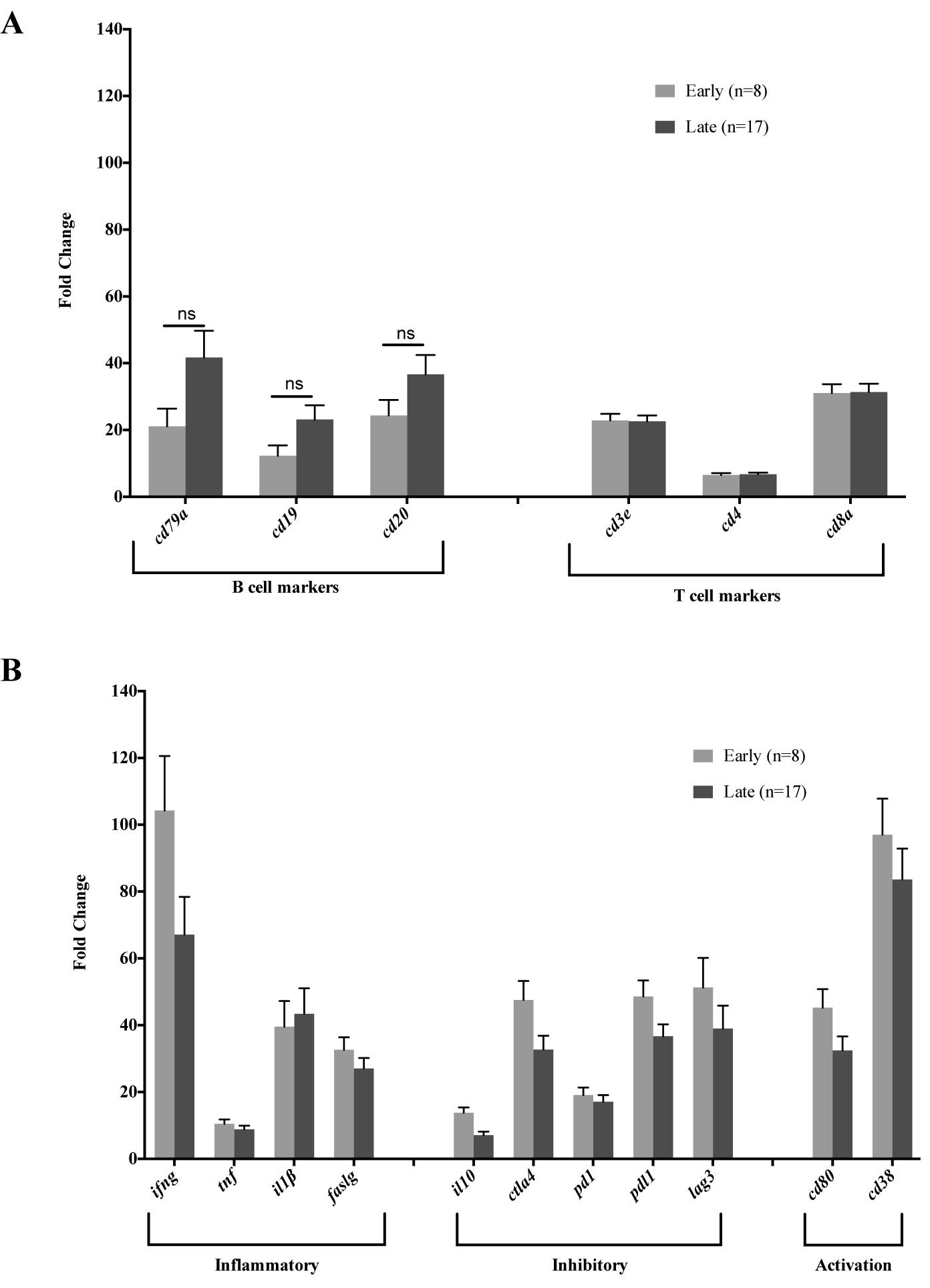

Supplement: S2 Fig — Bars represent the fold change (mean plus SEM) of RNA expression of cell-specific markers in early (light grey) and late (dark grey) cutaneous samples, each compared to healthy patients, showing (A) infiltration of B cell (cd79a, cd19, cd20) and T cell (cd3e, cd4, cd8a) biomarkers, and (B) increased inflammatory (ifnγ, tnf, IL12p40, IL-10, faslg), anti-inflammatory and inhibitory signals (il10, ctla4, pdcd1, cd274, lag3), and activation markers (cd80, cd38) in lesions with no difference observed between early and late. NS = not significant. (TIF) [file pntd.0004992.s002.tif]

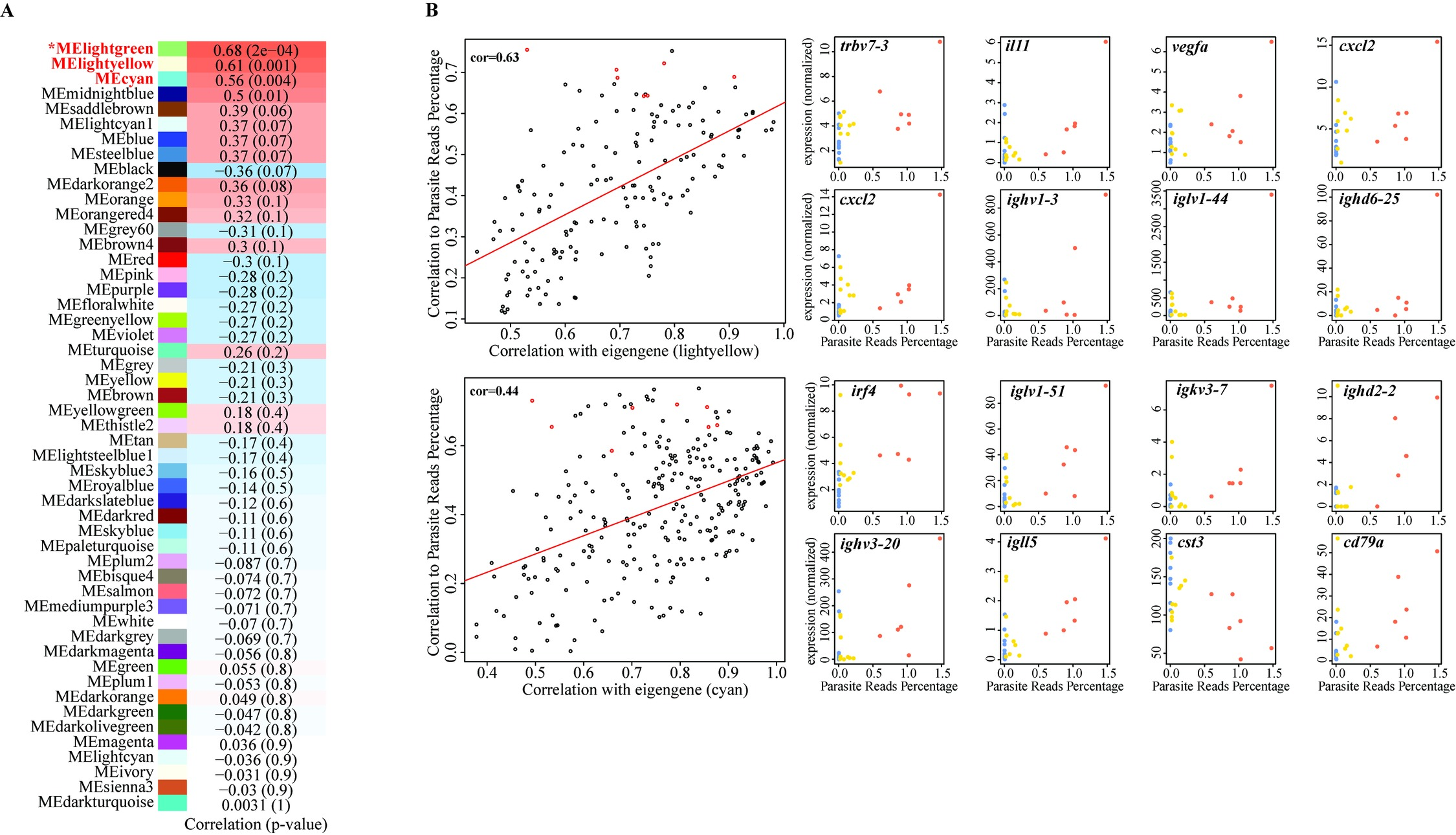

Supplement: S3 Fig — (A) Heatmap comparing the module eigengene relationship to the trait of percent parasite reads. Heatmap color indicates correlations of -1 (blue) to +1 (red) and p-values are shown in parentheses. Modules are sorted top to bottom by significance and each module is represented by a different color block. The three most significant modules are indicated in red type with the most significant module, indicated by a *, which is explored further in Fig 3C. (B) The second (light yellow, top) and third (cyan, bottom) module (by p-value) and representative genes from within the module correlated to percent parasite reads using WGCNA analysis. The left plots demonstrate module membership versus correlation to parasite percent reads for each gene. On the right, plots demonstrate normalized expression (rpkm) versus parasite percent reads for selected genes from within the modules (highlighted in red in the left plots). (TIF) [file pntd.0004992.s003.tif]

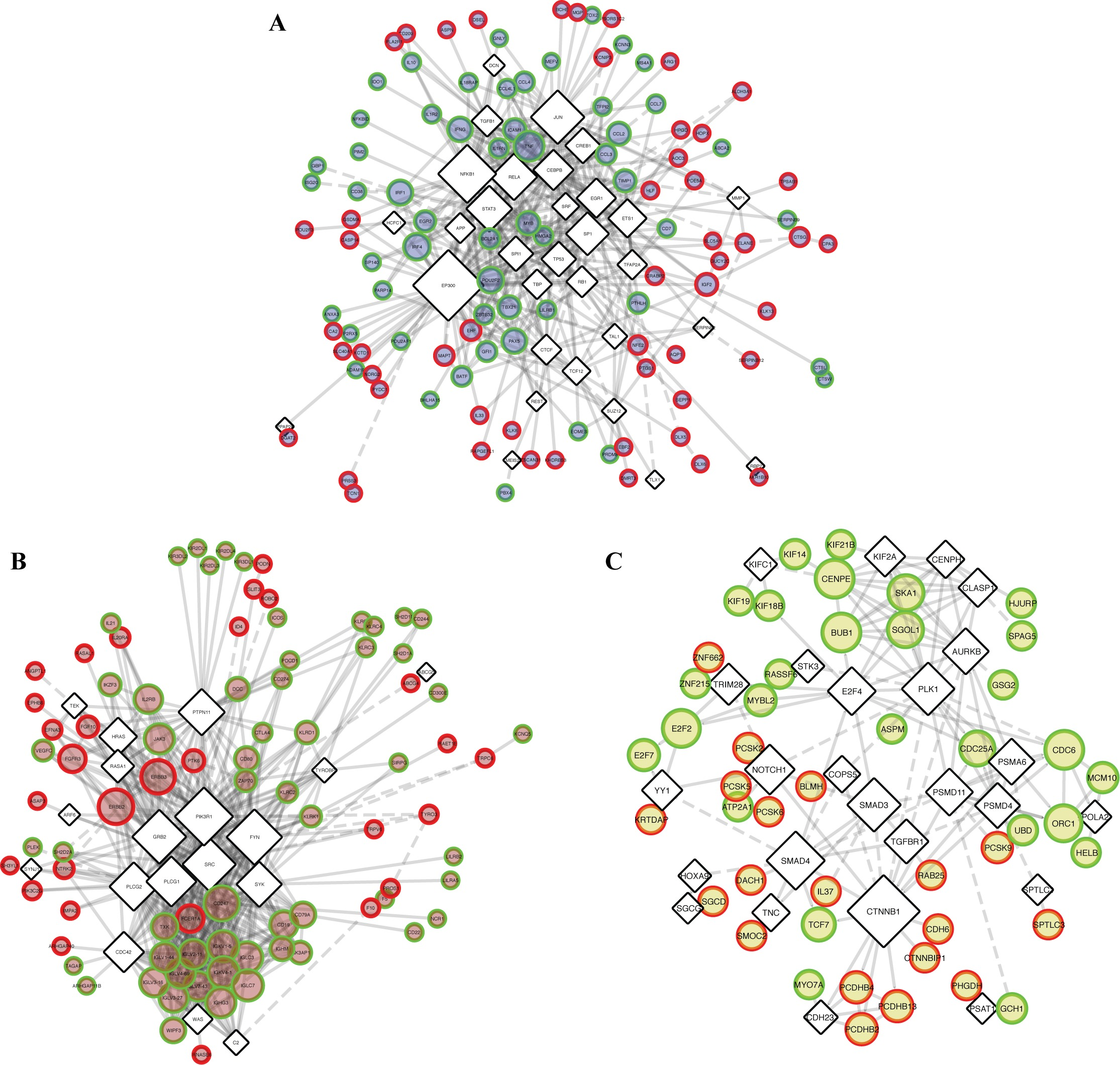

Supplement: S4 Fig — The largest three clusters consisting of 137 (A), 103 (B), and 72 (C) genes show numerous functional interactions (edges). Gene clusters are depicted by color and direction of differential expression is depicted by node border, with green borders designating upregulated and red borders designating downregulated genes. Node size indicates the number of interactions. (A) Network of genes associated with immune cell activation, costimulation, and cytokine and chemokine signaling, including TNF, IL-10, and multiple C-C motif chemokines regulated by NFκβ, CREB/STAT3, JUN, and SP1 signaling. (B) Network of 103 genes involved in B and T cell activation and inhibition, regulated by SYK, SRC, FYN, PLCG1 and 2, and PTPN11. (C) Interaction of 72 genes associated with cell growth and proliferation signals. (TIF) [file pntd.0004992.s004.tif]

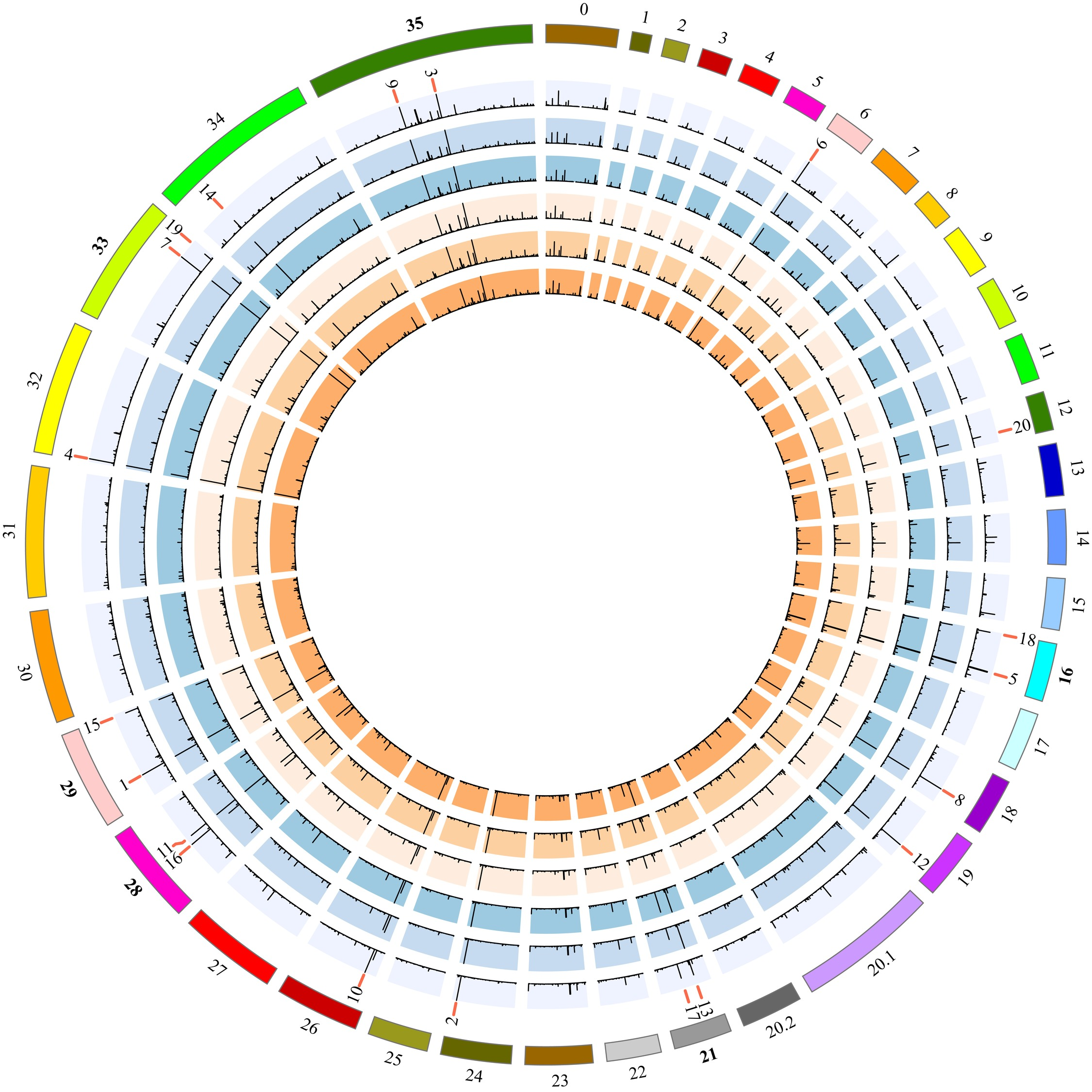

Supplement: S5 Fig — Representation of the L. braziliensis genome with the outermost ring (labeled 1–35) showing each individual chromosome. The subsequent concentric circles represent each of the six PTPos patient samples (3 early cutaneous shown in blue, 3 late cutaneous in orange). Vertical lines designate the relative expression levels of L. braziliensis gene expression in RPKM with the top 20 most highly-expressed genes marked by a red line, and numbered from 1–20. Chromosomes labeled in bold are viewed closer in Fig 5. (TIF) [file pntd.0004992.s005.tif]

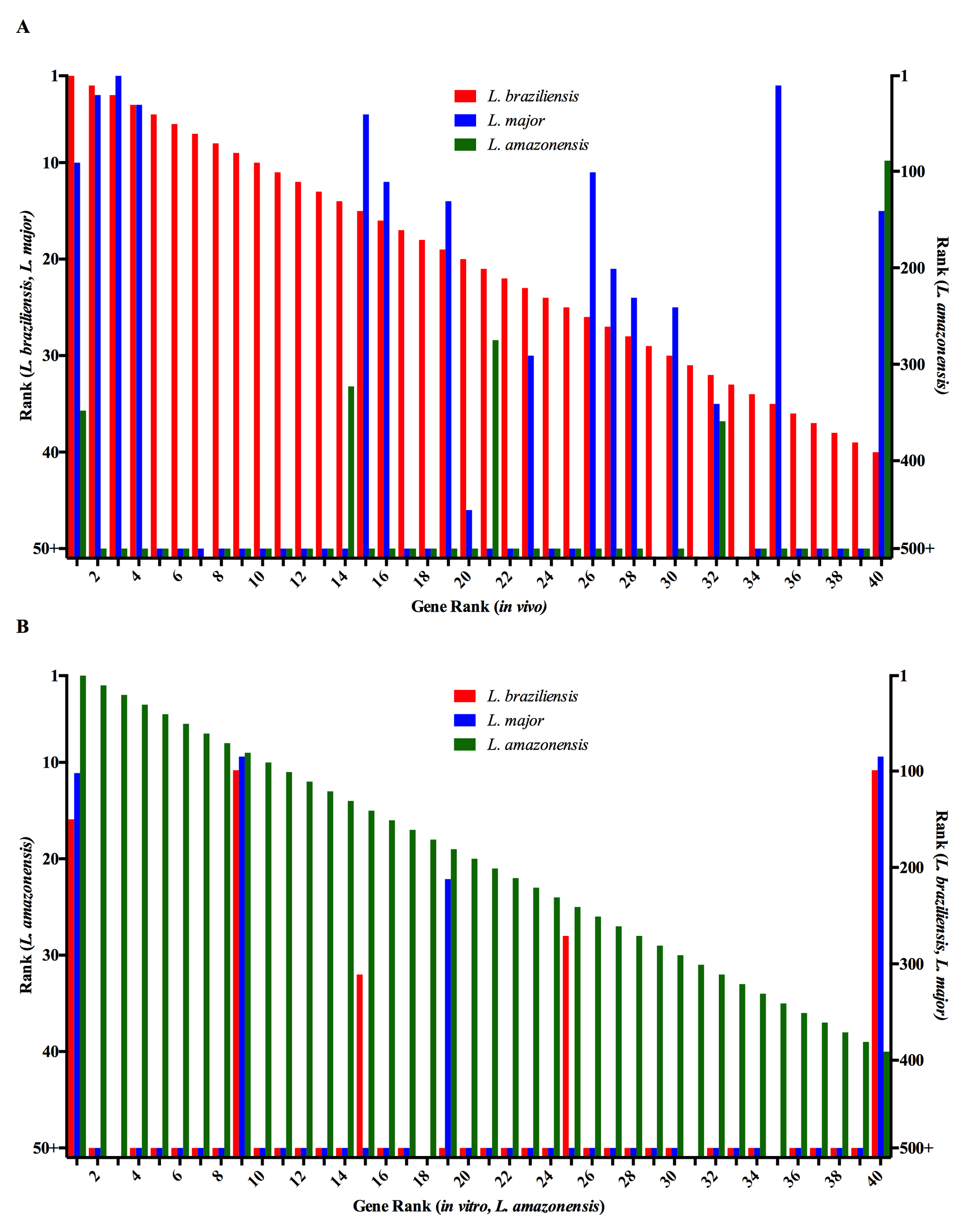

Supplement: S6 Fig — RPKM values of all samples were scaled to the sample with the highest total normalized reads. (A) The top 40 L. braziliensis genes (red) expressed in vivo as ranked by average RPKM (left axis) matched to the ranking of their homologous genes in L. major (blue, left axis, 1–50+) and L. amazonensis (green, right axis, 1–500+) expressed during in vitro infection of human macrophages at 72 hours post-infection. (B) The top 40 L. amazonensis genes (green) expressed during in vitro infection of human macrophages at 72 hours post-infection as ranked by average RPKM (left axis) matched to the ranking (1–500+) of their homologous genes in L. braziliensis (red, right axis) expressed during in vivo and L. major (blue, right axis) expressed during in vitro infection of human macrophages at 72 hours post-infection. (TIF) [file pntd.0004992.s006.tif]
